# Indications of musculoskeletal health in deceased male individuals with lower-limb amputations: comparison to non-amputee and diabetic controls

**DOI:** 10.1038/s41598-023-34773-w

**Published:** 2023-05-31

**Authors:** M. G. Finco, Caitlyn Finnerty, Wayne Ngo, Rachel A. Menegaz

**Affiliations:** 1 Department of Physiology and Anatomy, University of North Texas Health Science Center, 3500 Camp Bowie Blvd, Fort Worth, TX USA; 2 Department of Health and Exercise Science, The College of New Jersey, 2000 Pennington Rd, Ewing, NJ USA; 3 Texas College of Osteopathic Medicine, University of North Texas Health Science Center, 3500 Camp Bowie Blvd, Fort Worth, TX USA

**Keywords:** Anatomy, Musculoskeletal system, Bone, Cartilage, Muscle, Skeleton

## Abstract

Individuals with lower-limb amputations, many of whom have type 2 diabetes, experience impaired musculoskeletal health. This study: (1) compared residual and intact limbs of diabetic and non-diabetic post-mortem individuals with amputation to identify structures vulnerable to injury, and (2) compared findings to diabetic and healthy control groups to differentiate influences of amputation and diabetes on musculoskeletal health. Postmortem CT scans of three groups, ten individuals each, were included: (1) individuals with transtibial or transfemoral amputations, half with diabetes (2) diabetic controls, and (3) healthy controls. Hip and knee joint spaces, cross-sectional thigh muscle and fat areas, and cross-sectional bone properties (e.g. area, thickness, geometry) were measured. Wilcoxon Signed-Rank and Kruskal–Wallis tests assessed statistical significance. Asymmetry percentages between limbs assessed clinical significance. Residual limbs of individuals with amputation, particularly those with diabetes, had significantly less thigh muscle area and thinner distal femoral cortical bone compared to intact limbs. Compared to control groups, individuals with amputation had significantly narrower joint spaces, less thigh muscle area bilaterally, and thinner proximal femoral cortical bone in the residual limb. Diabetic individuals with amputation had the most clinically significant asymmetry. Findings tended to align with those of living individuals. However, lack of available medical information and small sample sizes reduced the anticipated clinical utility. Larger sample sizes of living individuals are needed to assess generalizability of findings. Quantifying musculoskeletal properties and differentiating influences of amputation and diabetes could eventually help direct rehabilitation techniques.

## Introduction

Forces acting on the human body during gait help maintain musculoskeletal health. Individuals with lower-limb amputation (IWAs) experience increased forces on the intact limb and reduced forces on the amputated, or residual, limb while walking with a prosthesis^1^. Chronic over or underuse of musculoskeletal structures alters patterns of musculoskeletal remodeling and can contribute to asymmetries between residual and intact limbs^2^. These asymmetries have been associated with adverse health outcomes such as increased risks of hip and knee osteoarthritis^2,3^, thigh muscle atrophy^4^, and femoral fracture^5^.

While gait asymmetries between residual and intact limbs have been studied extensively in this population, little attention has been paid to examining underlying structural musculoskeletal adaptations that have been associated with gait deviations^2^. Understanding structural musculoskeletal properties in this population could help reduce these risks by improving rehabilitation foci and improving clinical screening metrics. Specifically, these risks may be assessed by quantifying structural musculoskeletal properties, such as narrower hip and knee joint spaces to indicate osteoarthritis^6^, reduced muscle mass and fat tissue area to indicate muscle atrophy^7^, and reduced cross-sectional bone properties and moment of inertia to indicate fracture risk^8^.

Type 2 diabetes is a common comorbidity of IWAs^9^ and is also associated with increased risks of hip and knee osteoarthritis, thigh muscle atrophy, and femoral fracture risk^10–12^. However, diabetic IWAs are underrepresented in musculoskeletal properties literature that used anatomical imaging^2,13^. Determining how lower-limb amputation and diabetes differentially influence these risks can help inform rehabilitation techniques according to diabetic status in this population. As healthcare technology improves, quantifying musculoskeletal properties by pathology could help clinicians assess typical levels of musculoskeletal health, and implement rehabilitation exercises to reduce risks of hip and knee osteoarthritis, thigh muscle atrophy, and femoral fracture.

Therefore, this study quantified influences of amputation and diabetes on musculoskeletal parameters indicative of hip and knee osteoarthritis, thigh muscle atrophy, and femoral fracture risk. Our first aim was to determine if significant differences exist between residual and intact limbs of IWAs. We hypothesized that residual limbs, compared to intact limbs, would have significantly wider hip and knee joint spaces, reduced thigh muscle mass, and reduced cross-sectional bone properties (e.g. area, thickness, geometry). Our second aim was to compare IWA findings to diabetic and healthy control groups to differentiate interactions between amputation and diabetes on musculoskeletal health. We hypothesized IWAs, compared to diabetic and healthy control groups, would have significantly narrower hip and knee joint spaces, reduced muscle mass, and reduced cross-sectional bone properties. Overall, we expected diabetic IWAs would show the most impaired musculoskeletal health, followed by non-diabetic IWAs, diabetic controls, then healthy controls.

## Methods

### Study population

Deidentified data from deceased individuals did not require ethics approval from the North Texas Regional Institutional Review Board, per the institution’s policy. Computed tomography (CT) scans from deceased individuals were obtained with permission from the New Mexico Decedent Image Database (NMDID)^14^ from June to October 2021. The NMDID’s IRB approval is publicly posted on their website^14^. The NMDID used a Phillips Brilliance Big Bore 16 slice CT scanner with Z-position accuracy of ± 0.25 mm and resolution of up to 24 Lp/cm. Additional specifications for lower-limb scans are detailed on their website. Three groups were included: transtibial or transfemoral IWAs (half with diabetes), diabetic controls without amputation, and healthy controls without amputation.

To find IWAs, our preliminary search criteria was narrowed to males 40–90 years of age with potentially relevant causes of death (e.g., “diabetes”, which is listed as a selection for cause of death in the database as determined by the medical examiner) or record of lower limb surgery, as indicated by the NMDID records. IWAs were included if they had limb loss upon visual inspection of scout images, and excluded if they had advanced decomposition (determined by the CT technologist per NMDID, from a scale of no decomposition to mild, moderate, and advanced). A total of 10 IWAs were found in the NMDID database as of October 10th, 2021. Presence or absence of diabetes for was determined by a diagnosis of type II diabetes listed in the medical history. Once sex, age, and BMI of all 10 IWAs were recorded, diabetic and healthy controls were identified that were sex, age, and BMI matched to each of the 10 IWAs. Healthy controls were excluded if they had: amputation, diabetes, advanced body decomposition, cancer, or an age or BMI outside the range of identified IWAs. Diabetic controls were excluded for any of the above criteria for healthy controls, except diabetes.

### Sample demographics

In total, thirty males 42–79 years of age with BMIs of 19.7–48.9 kg/m^2^ were included. Summary demographics are provided in Table 1. BMIs for IWAs were adjusted according to equations in previous literature^15^. IWAs were added to the study until no more could be found, which resulted in ten IWAs (6 transtibial, 4 transfemoral). Five IWAs had a diagnosis of diabetes, and five did not have a diagnosis of diabetes. Therefore, ten diabetic controls and ten healthy controls were included in this study. Two IWAs were affected bilaterally. For these individuals, the residual limb refers to the most proximally amputated limb and the intact limb refers to the most distally amputated limb. Limitations regarding sample size, individuals affected bilaterally, and available medical history information are discussed in “Limitations and future work”. Supplemental Tables 1 and 2 contain data from each IWA, classified by level of amputation and whether they were affected unilaterally or bilaterally. Supplemental Tables 3–7 present all summarized data and p values.

**Table 1:** Summarized demographics.

	Sex	Age (years)	BMI (kg/m^2^)	Time from death to scan (days)	Race	Ethnicity	Primary cause of death	Side/Level of Amputation
Healthy controls (n = 10)	All M	57.50 ± 11.17	30.93 ± 8.05	61.20 ± 130.42	All White	4 of 10 Hispanic, Latino, or Middle Eastern	All natural	N/A
Diabetic controls (n = 10)	All M	54.00 ± 9.18	29.22 ± 6.89	26.20 ± 38.71	9 White; 1 Native American	2 of 10 Hispanic, Latino, or Middle Eastern	All diabetes	N/A
All IWAs (n = 10)	All M	61.90 ± 11.05	33.80 ± 8.31	23.11 ± 15.83	7 White; 1 Black or African American; 1 Hispanic; 1 Native American	5 of 10 Hispanic, Latino, or Middle Eastern	5 diabetes; 2 natural; 3 sepsis	5 unilateral TT; 3 unilateral TF; 1 bilateral TT; 1 bilateral TF
Diabetic IWAs (n = 5)	All M	67.00 ± 11.00	32.58 ± 8.81	14.00 ± 4.24	3 White; 1 Hispanic; 1 Native American	1 of 5 Hispanic, Latino, or Middle Eastern	2 natural; 3 sepsis	2 unilateral TT; 1 bilateral TT; 1 bilateral TF; 1 unilateral TF
Non-diabetic IWAs (n = 5)	All M	56.80 ± 9.42	35.01 ± 8.61	30.40 ± 18.20	1 Black or African American; 4 White	0 of 5 Hispanic, Latino, or Middle Eastern	All diabetes	3 unilateral TT; 2 unilateral TF;
*p *values between Healthy controls, diabetic controls, and All IWAs		0.18	0.98	0.08				

Summarized demographic characteristics of all individuals included in this study as reported in the New Mexico Decedent Image Database. Data depicted as means ± standard deviations. Differences among groups between age, BMI, and time from death to scan were calculated using a Kruskal–Wallis test. All *p* values are two-tailed, with significance set at 0.05. *IWAs* individuals with lower-limb amputation, *TT* transtibial, *TF* transfemoral, *N/A* not applicable.

### Joint space, tissue area, and femur morphology

CT scans were imported into 3D Slicer software (version 4.10) for the following bilateral measurements^16^. Author CF measured all musculoskeletal properties in the IWAs. Authors CF and WN were each randomly assigned to measure all musculoskeletal properties in half of the diabetic controls and half of the healthy controls.

Hip and knee joint spaces were measured from femoral head to acetabulum, and from femoral to tibial condyles, respectively. Fourteen measurements were averaged per hip and knee joint space, divided equally between anterior and posterior aspects or medial and lateral aspects, respectively, to provide a more accurate representation of the entire knee joint space.

Cross-sectional thigh muscle and fat tissue areas were measured at the femoral midshaft, defined as 50% of overall femur length. Within the midshaft slice, muscle and fat were identified using the threshold function within the Segmentation Editor module. Tissue areas were calculated using the Segmentation Statistics module.

Femur lengths were measured from greater trochanter to lateral condyle in intact limbs, and from greater trochanter to the lateral aspect of the distal end in residual limbs. Femoral head diameters were measured from anterior to posterior aspects and medial to lateral aspects. These two dimensions were averaged and expressed as a ratio of anterior–posterior to medial–lateral diameter. Femoral neck widths were measured superoinferiorly at the narrowest aspect. Femoral diaphysis width was measured anteroposteriorly at the midshaft.

### Femur geometry

Cross-sectional bone properties were also measured for the femur. CT scan slices were loaded into the BoneJ plugin (version 2) for Fiji to quantify cortical bone thickness and moment of inertia^17^. Three slices per femur were used: the proximal (25% of total femoral length), middle (50%), and distal (75%) femoral shaft. The slice was cropped to enclose the cortical bone and converted into an 8-bit image. The grayscale threshold range was adjusted to encompass the bone while excluding air and soft tissue. Threshold values were imputed into the Slice Geometry module of BoneJ to calculate biomechanical properties of the bone at the specified cross-section.

### Repeatability study

A repeatability study was conducted to ensure intra and inter-observer reliability between CF and WN’s control group measurements. CF and WN were assigned the same three scans, and collected all measurements three times with at least 24 h between each collection. Intra- and inter-observer reliability of CF and WN were determined using paired and unpaired t-tests, respectively. Percent differences in measurements were also calculated. No significant differences were found, and all reliability measurements differed by less than 10%.

### Statistics

NCSS Statistical Software (2021, LCC, Kaysville, UT, USA) was used for statistical analysis^18^. Missing data points (e.g. knee joint space absent due to transfemoral amputation) were not included. For Aim 1, Wilcoxon Signed-Rank tests assessed differences between residual and intact limbs of IWAs, right and left limbs of diabetic controls, and right and left limbs of healthy controls. For Aim 2, Kruskal–Wallis tests with Bonferroni corrections assessed differences between groups. Right and left limbs were averaged for diabetic and healthy controls to provide a single limb value. Two Kruskal–Wallis tests were performed: (1) compared the intact limbs of IWAs to diabetic and healthy controls, and (2) compared the residual limbs of IWAs to diabetic and healthy controls. Mann–Whitney p values were performed for significant Kruskal Wallis group comparisons. All p values are reported as two-tailed, with significance levels set at α ≤ 0.05.

### Calculation of asymmetry

Asymmetry between limbs was calculated to assess clinical significance. A threshold of 10% asymmetry is considered a clinically relevant threshold, where 0% is perfectly symmetrical and 100% is perfectly asymmetrical^13^. Asymmetry percentages were calculated using the following equation:

1 $$\mathrm{abs}\left(\left(\frac{I- R}{(I+R)\times 0.5}\right)\times 100\right)$$

For IWAs, I represents the intact limb value and R represents the residual limb value. For diabetic and healthy controls, the left limb value replaced I and the right limb value replaced R.

## Results

### Joint space, tissue area, and femur morphology

#### Between-limb comparisons

**Figure 1:**
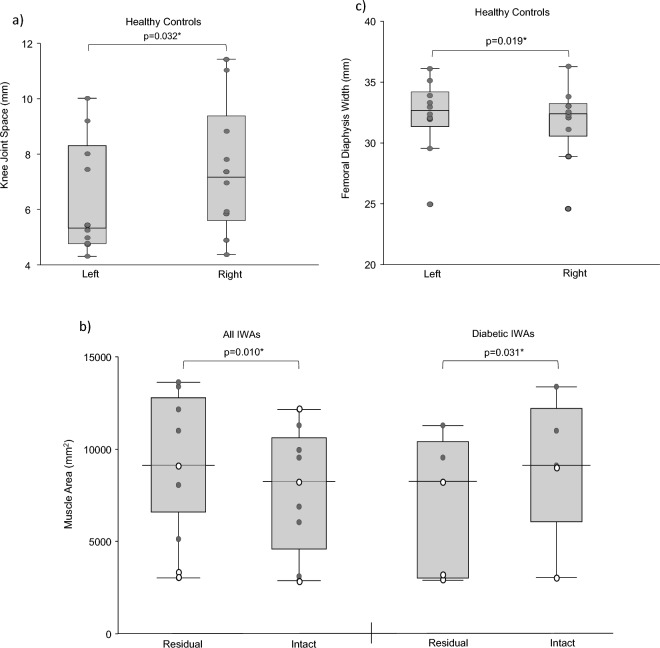
Between-limb comparisons with statistical significance for: (**a**) joint space, (**b**) tissue area, and (**c**) femur morphology. Dark grey points indicate data from transtibial participants, and white data points indicate data from transfemoral participants.

Data are presented in Fig. 1 and Supplemental Table 3. Healthy controls had significantly different knee joint spaces (*p* = 0.032) between right and left limbs. IWAs had significantly less muscle tissue area (*p* = 0.010) in residual limbs compared to intact limbs. Diabetic IWAs also showed this significance in muscle tissue area (*p* = 0.031). Diabetic controls had significantly different anterior–posterior femoral head width between right and left limbs (*p* = 0.014), and significant differences in femoral head ratio (*p* = 0.032). Healthy controls had significantly different femoral diaphysis widths (*p* = 0.019) between right and left limbs.

Healthy controls had clinically significant knee joint space asymmetry (14.85%). Non-diabetic IWAs had clinically significant hip (21.01%) and knee (17.90%) joint space asymmetry. IWAs had clinically significant muscle area (18.69%) asymmetry, higher in diabetic (26.09%) compared to non-diabetic (10.63%) IWAs.

#### Between-group comparisons

**Figure 2:**
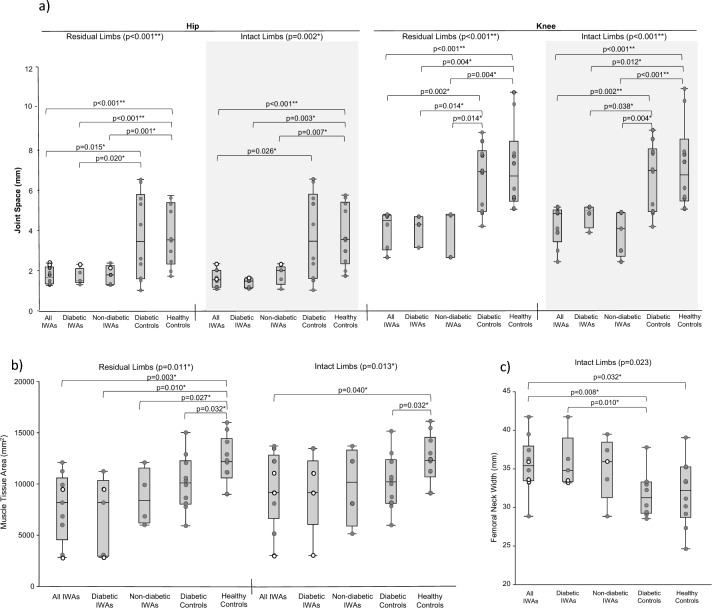
Between-group comparisons with statistical significance for: (**a**) joint space, (**b**) tissue area, and (**c**) femur morphology. Dark grey points indicate data from transtibial participants, and white data points indicate data from transfemoral participants.

Data are presented in Fig. 2 and Supplemental Table 4. Mann–Whitney post hoc comparisons for significant differences are presented in Supplemental Table 5. Compared to diabetic and healthy controls, IWAs had significantly narrower hip joint space on intact (*p* = 0.002) and residual (*p* < 0.001) limbs, narrower knee joint space on intact (*p* < 0.001) limbs, and wider femoral diaphysis on the intact limb (*p* = 0.023). Compared to healthy controls, IWAs had significantly narrower knee joint space on residual (*p* < 0.001) limbs, and reduced muscle area on intact (*p* = 0.013) and residual (*p* = 0.011) limbs.

### Femur geometry

#### Between-limb comparisons

**Figure 3:**
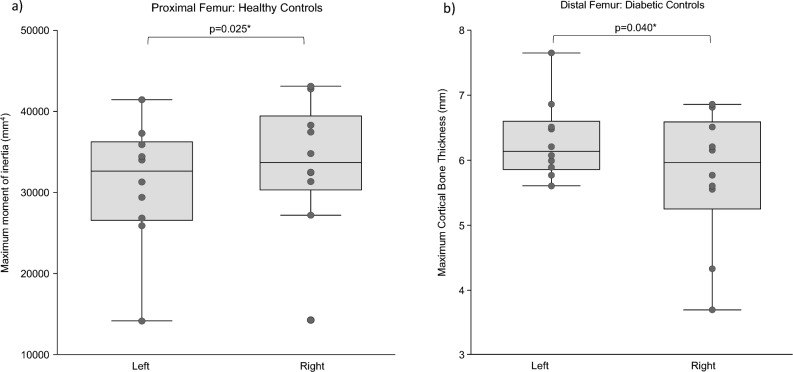
Between-limb comparisons with statistical significance for: (**a**) proximal femur geometry, and (**b**) distal femur geometry.

Data are presented in Fig. 3 and Supplemental Table 5. IWAs had significantly higher standard deviation cortical bone thickness (*p* = 0.037), which indicates higher variability in cortical bone thickness, at the femoral midshaft in residual limbs compared to intact limbs. Healthy controls had significantly different maximum moments of inertia (*p* = 0.025) at the proximal femur between right and left limbs. Diabetic controls had significantly different maximum (*p* = 0.040) and standard deviation (*p* = 0.025) thicknesses at the distal femur between right and left limbs.

At the proximal femur, non-diabetic IWAs had clinically significant cross-sectional area (10.37%), maximum (10.39%), and mean (12.41%) cortical bone thickness asymmetry. At the femoral midshaft, IWAs and diabetic IWAs had clinically significant standard deviation cortical bone thickness asymmetry (16.80% and 28.49%, respectively). At the distal femur, IWAs and non-diabetic IWAs had clinically significant standard deviation cortical bone thickness asymmetry (10.87% and 21.53%, respectively). Further, at the distal femur, diabetic IWAs had clinically significant cross-sectional area (15.15%), mean (16.13%), and standard deviation (11.73%) cortical thickness asymmetry. Additionally, non-diabetic IWAs had clinically significant asymmetry in minimum (10.53%) and maximum (17.15%) moments of inertia.

#### Between-group comparisons

**Figure 4:**
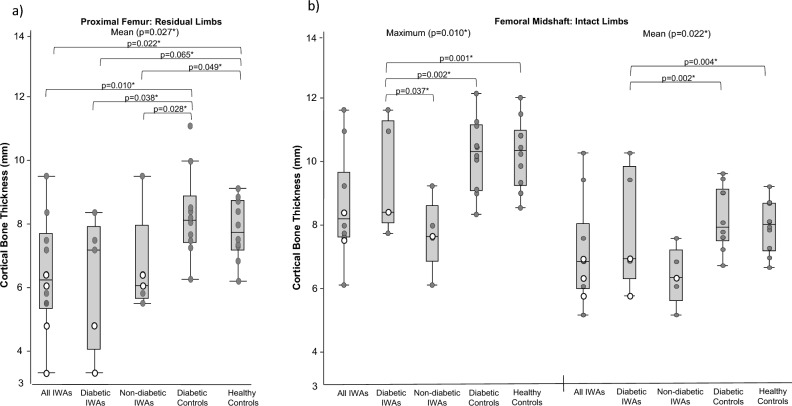
Between-group comparisons with statistical significance for: (**a**) proximal femur geometry, and (**b**) femoral midshaft geometry. Dark grey points indicate data from transtibial participants, and white data points indicate data from transfemoral participants.

Data are presented in Fig. 4 and Supplemental Table 6. At the proximal femur, residual limbs of IWAs had significantly narrower mean cortical thickness (*p* = 0.027) than diabetic and healthy controls. At the femoral midshaft, residual limbs of diabetic IWAs had significantly narrower maximum (*p* = 0.004) and mean (*p* = 0.017) cortical thickness than diabetic and healthy controls. At the distal femur, residual limbs of non-diabetic IWAs had significantly higher standard deviation cortical thickness (*p* = 0.020) than all groups.

## Discussion

Obtaining large cohorts of CT scans in living individuals with amputations can be challenging. Using the NMDID to collect musculoskeletal data from deceased individuals with amputations could help inform which properties might be most beneficial to examine in living individuals. To our knowledge, this was the first study to use CT scans to quantify musculoskeletal health in individuals with amputation, compare findings to diabetic and healthy controls, and is of the largest sample sizes to examine musculoskeletal health from an anatomical perspective in individuals with amputation. Our findings aligned with those from other musculoskeletal methodologies, (e.g. MRI, ultrasound) as well as known risk factors in living individuals^2^. Residual limbs of IWAs had higher indications of thigh muscle atrophy and distal femoral fracture risk compared to intact limbs. Compared to control groups, IWAs had higher indications of muscle atrophy and osteoarthritis bilaterally, along with proximal femoral fracture risk on the residual limb. Intact limbs of IWAs had wider femoral necks, indicative of increased femoral loading compared to diabetic and healthy controls. Diabetic IWAs tended to show the most impaired musculoskeletal health. Findings align with previous literature surrounding risk factors of hip and knee osteoarthritis, thigh muscle atrophy, and femoral fracture risk in living individuals with amputation^1–3^. However, major limitations include lack of healthcare information in the database related to amputation and prosthesis use, as well as our small sample size. Future research could examine larger sample sizes of living individuals to determine if findings are generalizable.

### Hip and knee osteoarthritis

Individuals who are unilateral lower-limb prosthesis users are more likely to develop hip and knee osteoarthritis on the intact limb, compared to the residual limb and nonamputee^1^. Prosthesis users rely on their intact limb, resulting in more biomechanical load, narrow joint space, and accelerated cartilage degeneration^1^. IWAs showed narrower hip and knee joint spaces bilaterally compared to controls, in line with prior work on the prevalence of osteoarthritis in the IWAs population compared to the general population^1^. However, in contrast with previous work^19,20^, hip or knee joint space were only significant between residual and intact limbs of non-diabetic IWAs. This may be due to our limited sample size.

### Thigh muscle atrophy

Thigh atrophy can hinder prosthetic control^21^ and balance^22^. IWAs, particularly those with diabetes, showed more thigh muscle atrophy in residual limbs compared to intact limbs. IWAs also showed indications of more thigh muscle atrophy bilaterally compared to diabetic and healthy controls. This aligns with work in living IWAs that used other methodologies (e.g. ultrasound, MRI)^4,23,24^. Elevated blood sugar levels, as seen in type 2 diabetes, may be associated with muscle atrophy^25^, which may explain why diabetic IWAs had the most thigh muscle atrophy. No significant differences in fat mass were found in any group, in contrast with previous work^23,26–28^, potentially due to our small sample sizes.

### Femoral fractures

Falls and fall-related injuries are prevalent in IWAs, so preventing fall-related fractures are of particular concern in this population^29,30^. In alignment with prior work, intact limbs of IWAs, compared to controls, had wider femoral necks and thinner cortical bone at the proximal femur^31,32^. Clinical significance among diabetic and non-diabetic IWAs suggest femoral adaptations may differ according to diabetic status. In nonamputees, proximal and distal femoral diaphysis are most adaptive to changes in biomechanical loading in nonamputees^33^. This may also occur in IWAs, as our findings and previous literature have shown thinner cortical bone and increased fracture rates at the proximal and distal femoral diaphysis in the residual limb^33,34^. This finding also supports previous literature regarding this population experiencing increased loading in the intact limb and decreased loading in the residual limb^1–3,32^. Proactive skeletal screening, rehabilitation exercises to promote residual limb loading, and earlier ambulation may help maintain skeletal health.

### Control groups

Leg dominance, combined with outliers or small sample size, may underlie significant differences between right and left limbs in healthy and diabetic control groups. Healthy controls had significant asymmetries between limbs in knee joint space and femoral diaphysis width, while diabetic controls had significant asymmetries between limbs in AP femoral head width and ratio. Additionally, healthy controls tended to have larger right-limb values for all cross-sectional data, and diabetic controls tended to have larger left-limb values for gross and cross-sectional data.

### Influence of diabetes

Diabetes, similar to amputation, has been associated with increased risks of developing sarcopenia, osteoarthritis, and osteopenia^10–12^. Despite having no statistical significance between diabetic and non-diabetic IWAs, diabetic IWAs showed the most clinical significance in hip and knee joint space as well as muscle tissue area. Diabetic IWAs also had the most impaired musculoskeletal health in several parameters, including knee joint space bilaterally, intact limb femoral neck width, and thinner intact femoral midshaft cortical bone. These trends suggest additive effects of amputation and diabetes on impaired musculoskeletal health, despite a lack of statistical significance. Clinicians should still consider other diabetic complications, such as retinopathy and peripheral neuropathy, which can lead to fall-related fractures^35^.

### Limitations and future work

A major limitation of this study was the amount of medical history information available from the NMDID. While potential confounding factors of musculoskeletal health were excluded (e.g. cancer, advanced body decomposition), information on prosthesis use was absent. For instance, all of the IWAs had a confirmed amputation surgery, but no record of time since amputation, diabetic onset, prosthesis use, or activity level. Therefore, musculoskeletal findings could not be assessed by these factors. This study included two individuals with bilateral amputations, as well as transtibial and transfemoral amputations, so findings should not be applied to one subset of amputation level.

Several properties on two individuals could not be measured, since the limb was outside the frame of the CT scan. Similarly, several femoral properties could not be measured in individuals with transfemoral amputation, particularly hip and knee joint space for one individual with bilateral hip replacements. Also, transfemoral residual limbs were measured at midshaft of the overall femur length. Further, while time between death and CT scan typically ranged between 10 and 30 days, two of the control individuals were scanned over 100 days after death. While hip joint replacements, transfemoral femur length, and time delay could theoretically have influenced findings, outliers were not observed in the data for these individuals.

The authors located, to the best of our knowledge, all available scans of IWAs in the database for this study. Future work can include a larger sample size to assess the generalizability of our findings as individuals are added to the database, compare our results to CT scans of living individuals, or examine individuals affected unilaterally or bilaterally separately. Additionally, application of findings are limited to males. Future work could examine effects of aging or include females, particularly to examine additive effects of osteoporosis risk due to hormonal changes during menopause.

## Conclusion

This study is currently among the largest sample sizes of musculoskeletal health in this population, and is the first to use CT scans of deceased individuals. While musculoskeletal health has been assessed in prior literature, few studies have collected anatomical properties underlying musculoskeletal health risks prevalent in this population. Findings tended to align with previously reported health risks in living individuals. Residual limbs of IWAs showed higher indications of muscle atrophy and fracture risk at the distal femur compared to intact limbs. IWAs, compared to control groups, showed higher indications of muscle atrophy and osteoarthritis bilaterally, as well as higher indications of fracture risk at the proximal femur on the residual limb. Proactive musculoskeletal screenings, targeted rehabilitation exercises, shortening time from amputation to ambulation, and ensuring optimal prosthetic fit and alignment could help clinicians reduce these risks. Musculoskeletal adaptations from amputation and diabetes tended to show an additive effect, but were not statistically significant. However, lack of available medical information and our small sample size greatly reduced the anticipated clinical utility of this study. Larger sample sizes and living individuals should be included in future work to assess generalizability of findings.

## Supplementary Information

Supplementary Information.

## Data Availability

All data can be made available upon request by contacting the corresponding author.
