# Supplementary material for: Indications of musculoskeletal health in deceased male individuals with lower-limb amputations: comparison to non-amputee and diabetic controls
Source: Sci Rep. 2023 May 31;13:8838. doi: 10.1038/s41598-023-34773-w (PMC10232508; doi:10.1038/s41598-023-34773-w)
Supplement: Supplementary file 1 — Supplementary Information. [file 41598_2023_34773_MOESM1_ESM.docx]

Supplemental Tables

**Supplemental Table 1: Amputee Data for Joint Space, Tissue Area, and Femur Morphology**

|  | **TTu** | **TTb** | **TTu** | **TFu** | **TTu** | **TFu** | **TFu** | **TTu** | **TFb** | **TTu** |
| --- | --- | --- | --- | --- | --- | --- | --- | --- | --- | --- |
| **Hip joint space most intact (mm)** | 2.9595 | 3.8237 | 4.4877 | 3.2324 | 2.5507 | 5.35815 | 2.06063 | 2.511 | 4.0997 | 3.5603 |
| **Hip joint space most comp (mm)** | 3.9307 | 4.1228 | 5.0457 | 3.3371 | 3.5678 | 4.6174 | 2.03967 | 2.7065 | 3.3001 | 3.0681 |
| **Knee Joint Space most intact (mm)** | 5.073 | 6.5995 | 2.3675 |  | 5.524 |  |  | 4.7018 |  | 6.2561 |
| **Knee Joint Space most comp (mm)** | 6.5359 | 4.543 | 4.116 |  | 5.7849 |  |  | 4.8853 |  | 5.7224 |
| **Femoral Head AP Diameter most intact (mm)** | 44.1 | 44.71 | 46.91 | 47.37 | 45.29 | 48.97 | 46.33 | 50.62 | 44.8 | 53 |
| **Femoral Head ML Diameter most intact (mm)** | 43.89 | 48.43 | 51.68 | 49.31 | 47.7 | 49.38 | 47.48 | 57.18 | 45.56 | 53.5 |
| **Femoral Head AP Diameter most comp (mm)** | 42.49 | 42.78 | 47.11 | 43.87 | 45.36 | 53.62 | 47.02 | 49.97 | 45.62 | 50.72 |
| **Femoral Head ML Diameter most comp (mm)** | 42.51 | 46.83 | 50.11 | 50.09 | 47.52 | 53.57 | 49.31 | 53.65 | 45.95 | 47.42 |
| **Femoral Head Ratio most intact (mm)** | 1.0048 | 0.9232 | 0.9077 | 0.9607 | 0.9495 | 0.9917 | 0.9758 | 0.8853 | 0.9833 | 0.9907 |
| **Femoral Head Ratio most comp (mm)** | 0.9995 | 0.9135 | 0.9401 | 0.8758 | 0.9545 | 1.0009 | 0.9536 | 0.9314 | 0.9928 | 1.0696 |
| **Fem Neck Width most intact (mm)** | 39.49 | 41.74 | 37.42 | 33.65 | 28.86 | 35.96 | 34.82 | 36.29 | 33.21 | 33.53 |
| **Fem Neck Width most comp (mm)** | 29.33 | 38.54 | 34.75 | 28.85 | 29.8 | 35.47 | 32.56 | 37.16 | 33.21 | 36.03 |
| **3D Fem Diaphysis Width most intact (mm)** | 28.71 | 36.05 | 54.08 | 31.84 | 28.18 |  | 31.74 | 34.9 | 29.56 | 33.35 |
| **3D Fem Diaphysis Width most comp (mm)** | 26.77 | 35.26 | 48.04 | 32.06 | 29.11 |  | 32.77 | 34.71 | 28.95 | 30.18 |
| **Muscle Area most intact (mm^2^)** | 13628 | 13392.5 | 5137.1 | 12154.6 | 8060.93 |  | 9116.17 | 9072.3 | 3019.12 | 11002.8 |
| **Muscle Area most comp (mm^2^)** | 12149.2 | 11290.1 | 6047.7 | 9959.22 | 6889.3 |  | 3117.56 | 8236.88 | 2890.7 | 9543.81 |
| **Fat Area most intact (mm^2^)** | 8729.33 | 6217.26 | 3221.35 | 9372.71 | 6839.44 |  | 4069.33 | 4055.02 | 1031.12 | 17261.3 |
| **Fat Area most comp (mm^2^)** | 12019.7 | 7303.15 | 3051.7 | 8602.14 | 6979.19 |  | 4516.6 | 4455.57 | 596.846 | 17398.7 |

Supplemental Table 1: IWA (amputee) data for joint space, tissue area, and femur morphology. Data depicted in means. Blank cells indicate data could not be collected. Abbreviations: TT= transtibial, TF=tranfemoral, u=unilateral, b=bilateral.

**Supplemental Table 2: Amputee Data for Femur Geometry**

|  |  | **TTu** | **TTb** | **TTu** | **TFu** | **TTu** | **TFu** | **TFu** | **TTu** | **TFb** | **TTu** |
| --- | --- | --- | --- | --- | --- | --- | --- | --- | --- | --- | --- |
| 25% | CSA most intact (mm^2) | 1348.4315 | 10922.7485 | 25218.5085 | 15555.859 | 2871.2635 | 9824.524 | 16006.9465 | 13872.6235 | 7905.245 | 16935.4635 |
|  | CSA most comp (mm^2) | 1201.29 | 10615.5155 | 30739.582 | 15841.484 | 2428.6015 | 7107.468 | 11050.224 | 13748.169 | 16245.876 | 15747.025 |
|  | Imin most intact (mm^4) | 388085.328 | 23079339.7 | 164995042 | 53862421.4 | 1262308.58 | 14048705.7 | 42850804.3 | 32645986 | 19699529 | 46946772.5 |
|  | Imin most comp (mm^4) | 371519.296 | 22866071.8 | 158925433 | 52602303.8 | 1154613.74 | 16244064.2 | 34258592.6 | 29904710.3 | 83111832.8 | 43511165.2 |
|  | Imax most intact (mm^4) | 324242.486 | 13770302.2 | 120818135 | 38040367.7 | 1221906.89 | 10425507.5 | 30054220.1 | 26023492.7 | 13610347.5 | 34939780.8 |
|  | Imax most comp (mm^4) | 296000.782 | 14978940.9 | 116780873 | 37154151.1 | 1094958.34 | 10940567.5 | 26508387.7 | 27766893.7 | 62937658.6 | 32759501.7 |
|  | Max thick most intact (mm) | 14.08 | 41.8995 | 49.055 | 43.363 | 22.8125 | 39.423 | 50.1735 | 48.826 | 28.4465 | 53.922 |
|  | Max thick most comp (mm) | 12.8365 | 41.326 | 61.126 | 41.135 | 19.6265 | 24.0115 | 33.5755 | 50.7995 | 36.706 | 44.48 |
|  | Mean thick most intact (mm) | 10.681 | 32.896 | 41.253 | 35.6015 | 17.427 | 34.9405 | 42.116 | 38.9865 | 20.848 | 43.0615 |
|  | Mean thick most comp (mm) | 9.457 | 31.9635 | 55.8795 | 36.892 | 14.36 | 19.3445 | 26.474 | 39.02 | 28.665 | 39.133 |
|  | SD thick most intact (mm) | 2.296 | 6.913 | 5.194 | 5.023 | 2.989 | 2.7315 | 4.4635 | 7.665 | 5.0985 | 5.592 |
|  | SD thick most comp (mm) | 2.458 | 6.7105 | 4.4215 | 3.9955 | 3.156 | 3.69 | 5.8675 | 7.845 | 6.624 | 3.3105 |
| 50% | CSA most intact (mm^2) | 2247.6955 | 9940.904 | 29193.4375 | 15967.369 | 2855.4655 | 9343.1855 | 13192.177 | 14139.1755 | 4227.3535 | 15883.155 |
|  | CSA most comp (mm^2) | 1491.5455 | 9979.9175 | 30488.053 | 16872.406 | 2354.876 | 8364.029 | 9708.8815 | 13950.3475 | 16748.444 | 19660.2285 |
|  | Imin most intact (mm^4) | 850819.629 | 18400770.1 | 154911268 | 44518372.2 | 1577136.24 | 10608820.9 | 32312962.7 | 32651839.1 | 12245951.4 | 40995242.9 |
|  | Imin most comp (mm^4) | 363128.33 | 20062275.4 | 156993494 | 48715575.9 | 1280744.29 | 15535218.8 | 25198555 | 30826524.9 | 55018461.4 | 61656121.3 |
|  | Imax most intact (mm^4) | 675764.995 | 14257054.6 | 103915902 | 40804524.6 | 839203.822 | 9116477.75 | 23304951.6 | 20093414.1 | 10519514.8 | 30441946.6 |
|  | Imax most comp (mm^4) | 291118.313 | 14993070.2 | 112672388 | 40811525.3 | 742177.625 | 10488409.7 | 18891934.5 | 19374755 | 40680212.5 | 47339243.1 |
|  | Max thick most intact (mm) | 18.956 | 43.5935 | 78.884 | 49.043 | 21.9825 | 45.565 | 48.321 | 56.5325 | 32.4435 | 53.1745 |
|  | Max thick most comp (mm) | 15.7815 | 36.115 | 63.4405 | 53.029 | 20.0675 | 29.1675 | 31.349 | 59.239 | 47.841 | 59.193 |
|  | Mean thick most intact (mm) | 14.9735 | 31.539 | 56.3715 | 38.8155 | 18.1555 | 38.4225 | 36.321 | 42.5775 | 28.331 | 42.315 |
|  | Mean thick most comp (mm) | 12.3865 | 28.9905 | 53.479 | 42.3175 | 15.102 | 24.3365 | 24.0615 | 43.969 | 43.082 | 47.6495 |
|  | SD thick most intact (mm) | 2.7715 | 6.5585 | 12.26 | 6.0835 | 2.9045 | 5.4155 | 7.066 | 8.446 | 3.61 | 6.153 |
|  | SD thick most comp (mm) | 2.4245 | 4.9 | 6.783 | 6.903 | 3.6465 | 3.0285 | 5.528 | 10.786 | 6.8235 | 7.0005 |
| 75% | CSA most intact (mm^2) | 1937.305 | 8456.7585 | 23084.318 | 10324.955 | 2375.321 |  |  | 11198.5205 |  | 9996.2795 |
|  | CSA most comp (mm^2) | 875.102 | 7822.244 | 22061.05 | 10718.8225 | 1613.905 |  |  | 10508.5375 |  | 18648.5435 |
|  | Imin most intact (mm^4) | 1500558.95 | 28982219.6 | 185590018 | 41712838.5 | 1426944.55 |  |  | 32470522.4 |  | 45598196.4 |
|  | Imin most comp (mm^4) | 342489.913 | 29364778.7 | 181735163 | 43687162.1 | 1072319.1 |  |  | 26897128.5 |  | 138613468 |
|  | Imax most intact (mm^4) | 1310090.73 | 22505591.7 | 140755833 | 33348990 | 1239514.98 |  |  | 29601497.6 |  | 36054177.6 |
|  | Imax most comp (mm^4) | 304809.927 | 20995722.1 | 136700520 | 37948385.1 | 825383.809 |  |  | 24464895 |  | 105804567 |
|  | Max thick most intact (mm) | 12.027 | 24.1745 | 39.472 | 27.656 | 15.623 |  |  | 31.8525 |  | 22.351 |
|  | Max thick most comp (mm) | 7.871 | 19.564 | 38.069 | 28.838 | 14.4655 |  |  | 29.673 |  | 34.5075 |
|  | Mean thick most intact (mm) | 8.8705 | 18.156 | 32.4285 | 20.8515 | 12.298 |  |  | 25.7675 |  | 18.2835 |
|  | Mean thick most comp (mm) | 5.748 | 16.3 | 30.6315 | 20.847 | 8.898 |  |  | 25.0335 |  | 27.418 |
|  | SD thick most intact (mm) | 1.646 | 2.746 | 4.449 | 4.1985 | 1.88 |  |  | 3.5275 |  | 1.931 |
|  | SD thick most comp (mm) | 1.416 | 1.7595 | 6.0905 | 4.32 | 3.264 |  |  | 2.7345 |  | 3.8955 |

Supplemental Table 2: IWA (amputee) data for femur geometry. Data depicted in means. Blank cells indicate data could not be collected. Abbreviations: TT= transtibial, TF=tranfemoral, u=unilateral, b=bilateral.

**Supplemental Table 3: Between-Limb Comparisons of Joint Space, Tissue Area, and Femur Morphology**

|  | **Hip Joint Space (mm)** | **Knee Joint Space (mm)** | **AP Femoral Head Width (mm)** | **ML Femoral Head Width (mm)** | **Femoral Head Ratio (AP/ML)** | **Femoral Neck Width (mm)** | **Femoral Diaphysis Width (mm)** | **Muscle Area (mm^2^)** | **Fat Area (mm^2^)** |
| --- | --- | --- | --- | --- | --- | --- | --- | --- | --- |
| **Healthy Controls (n=10)** |  |  |  |  |  |  |  |  |  |
| Left | 3.80 ± 1.66 | 6.41 ± 2.07 | 46.46 ± 4.66 | 46.78 ± 4.51 | 0.99 ± 0.04 | 31.76 ± 4.03 | 32.23 ± 3.13 | 12530.90 ± 2655.17 | 8999.39 ± 7546.05 |
| Right | 3.77 ± 1.47 | **7.44 ± 2.40*** | 46.15 ± 3.98 | 47.86 ± 5.36 | 0.97 ± 0.04 | 31.99 ± 4.62 | **31.72 ± 3.13*** | 12182.84 ± 2339.91 | 8964.95 ± 6950.44 |
| Direction of Asymmetry | L > R | L< R | L > R | L< R | L > R | L< R | L > R | L > R | L > R |
| Asymmetry | 0.69% | **14.85%** | 0.67% | 2.29% | 2.69% | 0.72% | 1.59% | 2.82% | 0.38% |
| **Diabetic Controls (n=10)** |  |  |  |  |  |  |  |  |  |
| Left | 3.65 ± 2.03 | 6.30 ± 1.77 | 43.22 ± 3.17 | 44.91 ± 4.20 | 0.96 ± 0.03 | 31.20 ± 2.94 | 31.98 ± 3.21 | 10295.51 ± 2445.50 | 9686.750 ± 6617.30 |
| Right | 3.80 ± 2.29 | 6.45 ± 2.16 | **44.84 ± 3.91*** | 45.32 ± 3.75 | **0.99 ± 0.02*** | 32.05 ± 2.98 | 32.24 ± 3.75 | 10052.81 ± 2989.30 | 9492.07 ± 6571.87 |
| Direction of Asymmetry | L< R | L< R | L< R | L< R | L< R | L< R | L< R | L > R | L > R |
| Asymmetry | 4.07% | 2.33% | 3.66% | 0.89% | 2.58% | 2.70% | 0.83% | 2.39% | 2.03% |
| **IWAs (n=10)** |  |  |  |  |  |  |  |  |  |
| Intact | 1.75 ± 0.42 | 4.02 ± 0.97 | 47.210 ± 2.88 | 49.41 ± 3.88 | 0.96 ± 0.04 | 35.50 ± 3.60 | 34.27 ± 7.90 | 9398.17 ± 3620.11 | 6755.21 ± 4758.96 |
| Residual | 1.59 ± 0.42 | 3.78± 0.94 | 46.86 ± 3.63 | 48.70 ± 3.41 | 0.96 ± 0.06 | 33.57 ± 3.40 | 33.09 ± 6.26 | **7791.61 ± 3333.84*** | 7213.73 ± 5059.76 |
| Direction of Asymmetry | Int > Res | Int > Res | Int > Res | Int > Res | Int < Res | Int > Res | Int > Res | Int > Res | Int < Res |
| Asymmetry | 9.56% | 5.93% | 0.75% | 1.46% | 0.61% | 5.58% | 3.48% | **18.69%** | 6.57% |
| **Diabetic IWAs (n=5)** |  |  |  |  |  |  |  |  |  |
| Intact | 1.79 ± 0.48 | 3.67 ± 1.10 | 46.53 ± 1.89 | 48.39 ± 2.89 | 0.96 ± 0.04 | 35.08 ± 4.08 | 35.70 ± 12.36 | 9745.16 ± 3870.95 | 7040.71 ± 2763.96 |
| Residual | 1.80 ± 0.49 | 3.79 ± 1.23 | 46.49 ± 4.34 | 48.76 ± 4.10 | 0.95 ± 0.05 | 31.64 ± 3.20 | 33.99 ± 9.61 | 8761.36 ± 2815.49 | 7663.18 ± 3723.55 |
| Direction of Asymmetry | Int < Res | Int < Res | Int > Res | Int < Res | Int > Res | Int > Res | Int > Res | Int > Res | Int < Res |
| Asymmetry | 0.37% | 4.83% | 0.08% | 0.76% | 0.91% | **10.30%** | 4.90% | **10.63%** | 8.47% |
| **Non-diabetic IWAs (n=5)** |  |  |  |  |  |  |  |  |  |
| Intact | 1.71 ± 0.41 | 4.51 ± 0.59 | 47.89 ± 3.73 | 50.43 ± 4.78 | 0.95 ± 0.05 | 35.92 ± 3.47 | 33.12 ± 2.57 | 9120.58 ± 3841.30 | 6526.81 ± 6278.47 |
| Residual | 1.38 ± 0.24 | 3.77 ± 0.87 | 47.22 ± 3.25 | 48.63 ± 3.06 | 0.97 ± 0.06 | 35.50 ± 2.56 | 32.37 ± 2.76 | **7015.81 ± 3819.82*** | 6854.17 ± 6359.25 |
| Direction of Asymmetry | Int > Res | Int > Res | Int > Res | Int > Res | Int < Res | Int > Res | Int > Res | Int > Res | Int < Res |
| Asymmetry | **21.01%** | **17.90%** | 1.41% | 3.63% | 2.13% | 1.17% | 2.28% | **26.09%** | 4.89% |

Supplemental Table 3: Between-limb comparisons of 3D Slicer data: joint space, tissue area, and femur morphology. Means with standard deviations. For healthy and diabetic control groups, right and left limbs were compared. For amputees, intact and residual limbs were compared. Wilcoxon-Signed Rank tests were used to assess significance. The significance level was set α ≤ 0.05. An asterisk (*) indicates a significant difference between limbs. Direction of asymmetry indicates which limb had greater mean values. Asymmetry indicates the amount of asymmetry between limbs where 0% is perfectly symmetrical and 100% is perfectly asymmetrical. Abbreviations: IWAs= individuals with lower-limb amputation, Int= intact limb, Res= residual limb, SD= standard deviation, AP= anterior-posterior, ML= medial-lateral.

**Supplemental Table 4: Between-Group Comparisons of Joint Space, Tissue Area, and Femur Morphology**

|  | **Hip Joint Space (mm)** | **Knee Joint Space (mm)** | **AP Femoral Head Width (mm)** | **ML Femoral Head Width (mm)** | **Femoral Head Ratio (AP/ML)** | **Femoral Neck Width (mm)** | **Femoral Diaphysis Width (mm)** | **Muscle Area (mm^2^)** | **Fat Area (mm^2^)** |
| --- | --- | --- | --- | --- | --- | --- | --- | --- | --- |
| **Healthy Controls (n=10)** |  |  |  |  |  |  |  |  |  |
| Right and Left Averaged | 3.78 ± 1.56 | 6.93 ± 2.24 | 46.31 ± 4.32 | 47.32 ± 4.93 | 0.98 ± 0.039 | 31.87 ± 4.32 | 31.98 ± 3.13 | 12356.87 ± 2497.54 | 8982.17 ± 7248.25 |
| **Diabetic Controls (n=10)** |  |  |  |  |  |  |  |  |  |
| Right and Left Averaged | 3.72 ± 2.16 | 6.37 ± 1.97 | 44.03 ± 3.54 | 45.11 ± 3.98 | 0.98 ± 0.02 | 31.62 ± 2.96 | 32.11 ± 3.48 | **10174.16 ± 2717.40†** | 9589.41 ± 6594.58 |
| **All IWAs (n=10)** |  |  |  |  |  |  |  |  |  |
| Intact | **1.75 ± 0.42†‡** | **4.02 ± 0.97*†‡** | 47.21 ± 2.88 | 49.41 ± 3.88 | 0.96 ± 0.04 | **35.50 ± 3.60†‡** | 34.27 ± 7.90 | **9398.17 ± 3620.11†‡** | 6755.21 ± 4758.96 |
| Residual | **1.59 ± 0.42*†‡** | **3.78± 0.94*†** | 46.86 ± 3.63 | 48.70 ± 3.41 | 0.96 ± 0.06 | **33.57 ± 3.40** | 33.09 ± 6.26 | **7791.61 ± 3333.84†‡** | 7213.73 ± 5059.76 |
| **Diabetic IWAs (n=5)** |  |  |  |  |  |  |  |  |  |
| Intact | 1.79 ± 0.48 | **3.62 ± 1.10*†‡** | 46.53 ± 1.89 | 48.39 ± 2.89 | 0.96 ± 0.04 | **35.08 ± 4.08†‡** | 35.70 ± 12.36 | **9745.16 ± 3870.95†‡** | 7040.71 ± 2763.92 |
| Residual | 1.80 ± 0.49 | 3.80 ± 1.24 | 46.49 ± 4.34 | 48.76 ± 4.10 | 0.95 ± 0.05 | 31.64 ± 3.20 | 34.00 ± 9.61 | 8761.36 ± 2815.49 | 7663.18 ± 3723.55 |
| **Non-diabetic IWAs (n=5)** |  |  |  |  |  |  |  |  |  |
| Intact | 1.71 ± 0.41 | 4.51 ± 0.59 | 47.89 ± 3.73 | 50.43 ± 4.78 | 0.95 ± 0.05 | **35.92 ± 3.47†‡** | 33.12 ± 2.57 | **9120.58 ± 3841.30†‡** | 6526.81 ± 6278.47 |
| Residual | **1.38 ± 0.24*†‡** | 3.77 ± 0.82 | 47.22 ± 3.25 | 48.63 ± 3.06 | 0.97 ± 0.06 | 35.50 ± 2.56 | 32.37 ± 2.76 | **7015.81 ± 3819.82†‡** | 6854.17 ± 6359.25 |
| ***p*-values (Healthy vs Diabetic vs Intact Limbs of All IWAs)** | **0.002*** | **< 0.001**** | 0.160 | 0.076 | 0.257 | 0.023* | 0.130 | **0.013*** | 0.387 |
| ***p*-values (Healthy vs Diabetic vs Residual of All IWAs)** | **< .001**** | **< .001**** | 0.250 | 0.134 | 0.324 | 0.130 | 0.488 | **0.011*** | 0.450 |

Supplemental Table 4: Between-group comparisons of 3D Slicer data for joint space, tissue area, and femur morphology. Means with standard deviations. For healthy and diabetic control groups, left and right limb values were averaged together. Kruskal-Wallis tests with Bonferroni corrections were used to assess significance. The significance level was set α ≤ 0.05. Bold text indicates p-values less than or equal to 0.05. Asterisks (*) indicate a p-value < 0.01, daggers (†) indicate significant differences compared to healthy controls, and double daggers (‡) indicate significant differences compared to diabetic controls. Abbreviations: IWAs= individuals with lower-limb amputation, SD= standard deviation; AP= anterior-posterior, ML= medial-lateral.

**Supplemental Table 5: Between-Limb Comparisons of Femur Geometry**

|  |  | **CSA (mm^2)** | **Imin (mm^4)** | **Imax(mm^4)** | **Max Thick 2d (mm)** | **Mean Thick 2d (mm)** | **SD Thick 2d (mm)** |
| --- | --- | --- | --- | --- | --- | --- | --- |
| **Proximal Femoral Shaft** | **Healthy Controls (n=10)** |  |  |  |  |  |  |
| **25%** | Left | 504.04 ± 72.67 | 41620.00 ± 10665.26 | 31075.98 ± 7636.79 | 9.03 ± 1.30 | 7.77 ± 0.78 | 0.95 ± 0.30 |
|  | Right | 518.22 ± 81.44 | 43648.46 ± 10596.43 | **33417.00 ± 8402.86*** | 9.21 ± 1.34 | 7.82 ± 0.95 | 0.95 ± 0.26 |
|  | Direction of Asymmetry | L < R | L < R | L < R | L < R | L < R | L > R |
|  | Asymmetry | 2.78% | 4.76% | 7.26% | 2.05% | 0.72% | 0.33% |
|  | **Diabetic Controls (n=10)** |  |  |  |  |  |  |
|  | Left | 534.63 ± 93.24 | 46211.27 ± 13618.75 | 33446.60 ± 10213.37 | 9.98 ± 2.03 | 8.25 ± 1.46 | 1.22 ± 0.46 |
|  | Right | 533.19 ± 110.94 | 46473.42 ± 16509.03 | 32798.82 ± 11755.50 | 10.15 ± 1.70 | 8.28 ± 1.38 | 1.39 ± 0.43 |
|  | Direction of Asymmetry | L > R | L < R | L < R | L < R | L < R | L < R |
|  | Asymmetry | 0.27% | 0.57% | 1.96% | 1.68% | 0.37% | **13.40%** |
|  | **IWAs (n=10)** |  |  |  |  |  |  |
|  | Intact | 511.98 ± 131.01 | 50951.37 ± 23625.27 | 39999.50 ± 18084.10 | 8.68 ± 1.55 | 7.09 ± 1.32 | 1.08 ± 0.33 |
|  | Residual | 476.20 ± 166.52 | 50354.88 ± 28305.25 | 37878.45 ± 18401.54 | 8.09 ± 1.97 | 6.44 ± 1.78 | 1.09 ± 0.27 |
|  | Direction of Asymmetry | Int > Res | Int > Res | Int > Res | Int > Res | Int > Res | Int < Res |
|  | Asymmetry | 7.24% | 1.18% | 5.45% | 6.98% | 9.60% | 0.60% |
|  | **Diabetic IWAs (n=5)** |  |  |  |  |  |  |
|  | Intact | 544.52 ± 171.81 | 55105.06 ± 32701.87 | 43929.45 ± 25212.90 | 9.08 ± 1.36 | 7.54 ± 1.49 | 0.99 ± 0.22 |
|  | Residual | 490.86 ± 201.76 | 52716.32 ± 39078.84 | 41062.19 ± 25648.81 | 8.19 ± 1.72 | 6.66 ± 1.63 | 1.01 ± 0.15 |
|  | Direction of Asymmetry | Int > Res | Int > Res | Int > Res | Int > Res | Int > Res | Int < Res |
|  | Asymmetry | **10.37%** | 4.43% | 6.75% | **10.39%** | **12.41%** | 2.26% |
|  | **Non-diabetic IWAs (n=5)** |  |  |  |  |  |  |
|  | Intact | 479.44 ± 80.33 | 46797.68 ± 11970.79 | 36069.56 ± 7843.88 | 8.27 ± 1.77 | 6.65 ± 1.10 | 1.18 ± 0.42 |
|  | Residual | 461.53 ± 145.42 | 47993.45 ± 16173.26 | 34694.70 ± 8870.45 | 7.99 ± 2.40 | 6.23 ± 2.10 | 1.17 ± 0.36 |
|  | Direction of Asymmetry | Int > Res | Int < Res | Int > Res | Int > Res | Int > Res | Int > Res |
|  | Asymmetry | 3.81% | 2.52% | 3.89% | 3.36% | 6.50% | 0.82% |
| **Middle Femoral Shaft** | **Healthy Controls (n=10)** |  |  |  |  |  |  |
| **50%** | Left | 492.86 ± 72.24 | 40155.28 ± 10382.12 | 29080.35 ± 8266.14 | 10.21 ± 0.91 | 8.02 ± 0.73 | 1.39 ± 0.28 |
|  | Right | 503.13 ± 81.09 | 41944.95 ± 11718.68 | 29984.94 ± 8619.12 | 10.47 ± 1.43 | 8.13 ± 0.87 | 1.44 ± 0.45 |
|  | Direction of Asymmetry | L < R | L < R | L < R | L < R | L < R | L < R |
|  | Asymmetry | 2.06% | 4.36% | 3.06% | 2.48% | 1.26% | 4.07% |
|  | **Diabetic Controls (n=10)** |  |  |  |  |  |  |
|  | Left | 525.26 ± 89.01 | 42877.95 ± 16659.48 | 30743.20 ± 10097.50 | 10.53 ± 1.15 | 8.49 ± 0.84 | 1.35 ± 0.45 |
|  | Right | 506.13 ± 98.12 | 41817.58 ± 16253.89 | 29336.44 ± 10457.54 | 10.06 ± 1.39 | 8.16 ± 1.18 | 1.33 ± 0.30 |
|  | Direction of Asymmetry | L > R | L > R | L > R | L > R | L > R | L > R |
|  | Asymmetry | 3.71% | 2.50% | 4.68% | 4.57% | 4.04% | 1.69% |
|  | **IWAs (n=10)** |  |  |  |  |  |  |
|  | Intact | 511.53 ± 154.98 | 48656.50 ± 30624.77 | 36209.66 ± 22120.00 | 8.64 ± 1.66 | 7.29 ± 1.52 | 1.13 ± 0.26 |
|  | Residual | 474.15 ± 169.90 | 46070.47 ± 32146.77 | 33372.45 ± 21112.44 | 9.03 ± 2.53 | 6.99 ± 1.81 | **1.33 ± 0.35*** |
|  | Direction of Asymmetry | Int > Res | Int > Res | Int > Res | Int < Res | Int > Res | Int < Res |
|  | Asymmetry | 7.59% | 5.46% | 8.16% | 4.39% | 4.14% | **16.80%** |
|  | **Diabetic IWAs (n=5)** |  |  |  |  |  |  |
|  | Intact | 555.07 ± 209.72 | 55105.68 ± 43469.35 | 41416.50 ± 30843.46 | 9.51 ± 1.75 | 7.98 ± 1.81 | 1.16 ± 0.20 |
|  | Residual | 514.22 ± 218.10 | 54975.60 ± 47994.16 | 39025.22 ± 31171.98 | 9.86 ± 2.58 | 7.36 ± 1.65 | 1.54 ± 0.37 |
|  | Direction of Asymmetry | Int > Res | Int > Res | Int > Res | Int < Res | Int > Res | Int < Res |
|  | Asymmetry | 7.64% | 0.24% | 5.95% | 3.66% | 8.09% | **28.49%** |
|  | **Non-diabetic IWAs (n=5)** |  |  |  |  |  |  |
|  | Intact | 467.99 ± 72.93 | 42207.32 ± 10800.85 | 31002.82 ± 9045.13 | 7.78 ± 1.12 | 6.60 ± 0.86 | 1.10 ± 0.33 |
|  | Residual | 442.08 ± 138.43 | 38946.37 ± 14018.11 | 28850.23 ± 10255.95 | 8.37 ± 2.57 | 6.70 ± 2.06 | 1.17 ± 0.26 |
|  | Direction of Asymmetry | Int > Res | Int > Res | Int > Res | Int < Res | Int < Res | Int < Res |
|  | Asymmetry | 5.69% | 8.04% | 7.19% | 7.28% | 1.55% | 6.26% |
| **Distal Femoral Shaft** | **Healthy Controls (n=10)** |  |  |  |  |  |  |
| **75%** | Left | 382.22 ± 58.28 | 48118.49 ± 14337.67 | 37968.37 ± 11143.22 | 5.55 ± 0.90 | 4.63 ± 0.59 | 0.61 ± 0.18 |
|  | Right | 390.46 ± 61.91 | 50445.11 ± 16873.66 | 39463.18 ± 12036.90 | 5.91 ± 0.83 | 4.73 ± 0.55 | 0.69 ± 0.14 |
|  | Direction of Asymmetry | L < R | L < R | L < R | L < R | L < R | L < R |
|  | Asymmetry | 2.13% | 4.72% | 3.86% | 6.29% | 2.11% | **10.87%** |
|  | **Diabetic Controls (n=10)** |  |  |  |  |  |  |
|  | Left | 397.60 ± 34.27 | 46510.46 ± 15166.72 | 38368.07 ± 15022.90 | 6.41 ± 0.63 | 5.07 ± 0.53 | 0.77 ± 0.19 |
|  | Right | 382.83 ± 69.19 | 46991.59 ± 16048.00 | 37397.42 ± 15990.31 | **5.84 ± 1.07*** | 4.78 ± 0.79 | **0.62 ± 0.17*** |
|  | Direction of Asymmetry | L > R | L < R | L > R | L > R | L > R | L > R |
|  | Asymmetry | 3.79% | 1.03% | 2.56% | 9.38% | 5.91% | **21.53%** |
|  | **IWAs (n=10)** |  |  |  |  |  |  |
|  | Intact | 390.91 ± 114.06 | 58415.07 ± 36624.33 | 45178.18 ± 26176.32 | 5.61 ± 1.00 | 4.52 ± 1.00 | 0.70 ± 0.18 |
|  | Residual | 374.98 ± 139.14 | 59347.39 ± 42489.22 | 47885.19 ± 31837.10 | 5.43 ± 1.05 | 4.29 ± 0.84 | 0.78 ± 0.24 |
|  | Direction of Asymmetry | Int > Res | Int < Res | Int < Res | Int > Res | Int > Res | Int < Res |
|  | Asymmetry | 4.16% | 1.58% | 5.82% | 3.18% | 5.15% | **10.20%** |
|  | **Diabetic IWAs (n=5)** |  |  |  |  |  |  |
|  | Intact | 408.84 ± 150.76 | 60167.10 ± 48199.65 | 47110.10 ± 35097.36 | 5.82 ± 1.26 | 4.82 ± 1.29 | 0.80 ± 0.15 |
|  | Residual | 351.26 ± 181.41 | 57004.36 ± 57054.68 | 45708.57 ± 43083.97 | 5.44 ± 1.17 | 4.10 ± 1.00 | 0.90 ± 0.16 |
|  | Direction of Asymmetry | Int > Res | Int > Res | Int > Res | Int > Res | Int > Res | Int < Res |
|  | Asymmetry | **15.15%** | 5.40% | 3.02% | 6.78% | **16.13%** | **11.73%** |
|  | **Non-diabetic IWAs (n=5)** |  |  |  |  |  |  |
|  | Intact | 368.50 ± 56.45 | 56225.05 ± 21628.47 | 42763.29 ± 13067.91 | 5.34 ± 0.67 | 4.15 ± 0.28 | 0.58 ± 0.14 |
|  | Residual | 406.59 ± 78.06 | 62471.43 ± 22528.35 | 50787.37 ± 15308.74 | 5.42 ± 1.11 | 4.56 ± 0.67 | 0.61 ± 0.24 |
|  | Direction of Asymmetry | Int < Res | Int < Res | Int < Res | Int < Res | Int < Res | Int < Res |
|  | Asymmetry | 9.83% | **10.53%** | **17.15%** | 1.52% | 9.29% | 5.81% |

Supplemental Table 5: Between-limb comparisons of BoneJ data for femur geometry. Means with standard deviations of the proximal, middle, and distal femoral shafts. For healthy and diabetic control groups, right and left limbs were compared. For amputees, intact and residual limbs were compared. Wilcoxon-Signed Rank tests were used to assess significance. The significance level was set α ≤ 0.05. An asterisk (*) indicates a significant difference between limbs. Direction of asymmetry indicates which limb had greater mean values. Asymmetry indicates the amount of asymmetry between limbs where 0% is perfectly symmetrical and 100% is perfectly asymmetrical. Abbreviations: IWAs= individuals with lower-limb amputation, Int= intact limb, Res= residual limb, CSA= cross-sectional area, Imin= minimum moment of inertia, Imax= maximum moment of inertia, Max= maximum, SD=standard deviation, Thick= cortical bone thickness.

**Supplemental Table 6: Between-Group Comparisons of Femur Geometry**

|  |  | **CSA (mm^2^)** | **Imin (mm^4^)** | **Imax(mm^4^)** | **Max Thick 2d (mm)** | **Mean Thick 2d (mm)** | **SD Thick 2d (mm)** |
| --- | --- | --- | --- | --- | --- | --- | --- |
| **Proximal Femoral Shaft** | **Healthy Controls (n=10)** |  |  |  |  |  |  |
| **25%** | Right and Left Averaged | 511.13 ± 77.05 | 42634.23 ± 10630.84 | 32246.49 ± 8019.83 | 9.119 ± 1.320 | 7.79 ± 0.87 | 0.95 ± 0.28 |
|  | **Diabetic Controls (n=10)** |  |  |  |  |  |  |
|  | Right and Left Averaged | 533.91 ± 102.09 | 46342.35 ± 15063.89 | 33122.710± 10984.43 | 10.07 ± 1.87 | 8.27 ± 1.42 | 1.30 ± 0.42 |
|  | **All IWAs (n=10)** |  |  |  |  |  |  |
|  | Intact | 511.98 ± 131.01 | 50951.37 ± 23625.27 | 39999.50 ± 18084.10 | 8.68 ± 1.55 | 7.09 ± 1.32 | 1.08 ± 0.33 |
|  | Residual | 476.20 ± 166.52 | 50354.88 ± 28305.25 | 37878.45 ± 18401.54 | 8.09 ± 1.97 | **6.44 ± 1.78†‡** | 1.09 ± 0.27 |
|  | **Diabetic IWAs (n=5)** |  |  |  |  |  |  |
|  | Intact | 544.52 ± 171.81 | 55105.06 ± 32701.87 | 43929.45 ± 25212.90 | 9.08 ± 1.36 | 7.54 ± 1.49 | 0.99 ± 0.22 |
|  | Residual | 490.86 ± 201.76 | 52716.32 ± 39078.84 | 41062.19 ± 25648.81 | 8.19 ± 1.72 | 6.66 ± 1.63 | 1.01 ± 0.15 |
|  | **Non-diabetic IWAs (n=5)** |  |  |  |  |  |  |
|  | Intact | 479.44 ± 80.33 | 46797.68 ± 11970.79 | 36069.56 ± 7843.88 | 8.27 ± 1.77 | 6.65 ± 1.10 | 1.18 ± 0.42 |
|  | Residual | 461.53 ± 145.42 | 47993.45 ± 16173.26 | 34694.70 ± 8870.45 | 7.99 ± 2.40 | 6.23 ± 2.10 | 1.17 ± 0.36 |
| **Middle Femoral Shaft** | **Healthy Controls (n=10)** |  |  |  |  |  |  |
| **50%** | Right and Left Averaged | 498.00 ± 76.67 | 41050.11 ± 11050.40 | 29532.64 ± 8442.63 | 10.34 ± 1.17 | 8.07 ± 0.80 | 1.42 ± 0.37 |
|  | **Diabetic Controls (n=10)** |  |  |  |  |  |  |
|  | Right and Left Averaged | 515.69 ± 93.56 | 42347.76 ± 16456.68 | 30039.82 ± 10277.51 | 10.30 ± 1.27 | 8.33 ± 1.01 | 1.34 ± 0.38 |
|  | **All IWAs (n=10)** |  |  |  |  |  |  |
|  | Intact | 511.53 ± 154.98 | 48656.50 ± 30624.77 | 36209.66 ± 22119.99 | 8.64 ± 1.66 | 7.29 ± 1.52 | 1.13 ± 0.26 |
|  | Residual | 474.15 ± 169.90 | 46070.47 ± 32146.77 | 33372.45 ± 21112.44 | 9.03 ± 2.53 | 6.99 ± 1.81 | 1.33 ± 0.35 |
|  | **Diabetic IWAs (n=5)** |  |  |  |  |  |  |
|  | Intact | 555.07 ± 209.72 | 55105.68 ± 43469.35 | 41416.50 ± 30843.46 | 9.51 ± 1.75 | 7.98 ± 1.81 | 1.16 ± 0.20 |
|  | Residual | 514.22 ± 218.10 | 54975.60 ± 47994.16 | 39025.22 ± 31171.98 | 9.86 ± 2.57 | 7.36 ± 1.65 | 1.54 ± 0.37 |
|  | **Non-diabetic IWAs (n=5)** |  |  |  |  |  |  |
|  | Intact | 467.99 ± 72.93 | 42207.32 ± 10800.85 | 31002.82 ± 9045.13 | **7.78 ± 1.12†‡** | 6.60 ± 0.86‡ | 1.10 ± 0.33 |
|  | Residual | 442.08 ± 138.43 | 38946.37 ± 14018.11 | 28850.23 ± 10255.95 | 8.37 ± 2.57 | 6.70 ± 2.06 | 1.17 ± 0.26 |
| **Distal Femoral Shaft** | **Healthy Controls (n=10)** |  |  |  |  |  |  |
| **75%** | Right and Left Averaged | 386.34 ± 60.01 | 49281.80 ± 15605.66 | 38715.78 ± 11590.06 | 5.73 ± 0.87 | 4.68 ± 0.57 | 0.65 ± 0.16 |
|  | **Diabetic Controls (n=10)** |  |  |  |  |  |  |
|  | Right and Left Averaged | 390.22 ± 51.73 | 46751.03 ± 15607.36 | 37882.75 ± 15506.61 | 6.13 ± 0.85 | 4.92 ± 0.66 | 0.69 ± 0.18 |
|  | **All IWAs (n=10)** |  |  |  |  |  |  |
|  | Intact | 390.91 ± 114.06 | 58415.07 ± 36624.33 | 45178.18 ± 26176.32 | 5.61 ± 1.00 | 4.52 ± 1.00 | 0.70 ± 0.18 |
|  | Residual | 374.98 ± 139.14 | 59347.39 ± 42489.22 | 47885.19 ± 31837.10 | 5.43 ± 1.05 | 4.29 ± 0.84 | 0.78 ± 0.24 |
|  | **Diabetic IWAs (n=5)** |  |  |  |  |  |  |
|  | Intact | 408.84 ± 150.75 | 60167.10 ± 48199.65 | 47110.09 ± 35097.36 | 5.82 ± 1.26 | 4.82 ± 1.29 | 0.80 ± 0.15 |
|  | Residual | 351.26 ± 181.41 | 57004.36 ± 57054.68 | 45708.57 ± 43083.92 | 5.44 ± 1.17 | 4.10 ± 1.00 | 0.90 ± 0.16 |
|  | **Non-diabetic IWAs (n=5)** |  |  |  |  |  |  |
|  | Intact | 368.50 ± 56.45 | 56225.05 ± 21628.47 | 42763.29 ± 13067.91 | 5.34 ± 0.67 | 4.15 ± 0.28 | 0.58 ± 0.14 |
|  | Residual | 406.60 ± 78.06 | 62471.43 ± 22528.35 | 50787.37 ± 15308.74 | 5.42 ± 1.11 | 4.56 ± 0.67 | **0.61 ± 0.24†‡** |

Supplemental Table 6: Between-group comparisons of BoneJ data for femur geometry. Means with standard deviations of the proximal, middle, and distal femoral shafts. For healthy and diabetic control groups, left and right limb values were averaged together. For amputees, intact and compromised limbs were compared separately. Kruskal-Wallis tests with Bonferroni corrections were used to assess significance. The significance level was set α ≤ 0.05. Bold text indicates p-values less than or equal to 0.05. Asterisks (*) indicate a p-value < 0.01, daggers (†) indicate significant differences compared to healthy controls, and double daggers (‡) indicate significant differences compared to diabetic controls. Abbreviations: IWAs= individuals with lower-limb amputation, CSA= cross-sectional area, Imin= minimum moment of inertia, Imax= maximum moment of inertia, Max= maximum, SD=standard deviation, Thick= cortical bone thickness.

**Supplemental Table 7: Mann-Whitney Comparisons**

| **Significant Parameter (Kruskal-Wallis *p*-value)** |  |  |  |  |  |
| --- | --- | --- | --- | --- | --- |
| **Hip Joint Space (p = 0.002)** | Healthy | Diabetic | All IWAs (Int) | Non-diabetic IWAs (Int) | Diabetic IWAs (Int) |
| Healthy |  |  |  |  |  |
| Diabetic | 0.398 |  |  |  |  |
| All IWAs (Int) | **< 0.001** | **0.026** |  |  |  |
| Non-diabetic IWAs (Int) | **0.007** | 0.065 | 0.4755 |  |  |
| Diabetic IWAs (Int) | **0.003** | 0.277 | 0.4755 | 0.4585 |  |
| **Hip Joint Space (p < 0.001)** | Healthy | Diabetic | All IWAs (Res) | Non-diabetic IWAs (Res) | Diabetic IWAs (Res) |
| Healthy |  |  |  |  |  |
| Diabetic | 0.398 |  |  |  |  |
| All IWAs (Res) | **< 0.001** | **0.015** |  |  |  |
| Non-diabetic IWAs (Res) | **0.001** | 0.083 | 0.212 |  |  |
| Diabetic IWAs (Res) | **< 0.001** | **0.020** | 0.212 | 0.111 |  |
| **Knee JoInt Space (p < 0.001)** | Healthy | Diabetic | All IWAs (Int) | Non-diabetic IWAs (Int) | Diabetic IWAs (Int) |
| Healthy |  |  |  |  |  |
| Diabetic | 0.290 |  |  |  |  |
| All IWAs (Int) | **< 0.001** | **0.002** |  |  |  |
| Non-diabetic IWAs (Int) | **< 0.001** | **0.004** | 0.251 |  |  |
| Diabetic IWAs (Int) | **0.012** | **0.038** | 0.219 | 0.143 |  |
| **Knee Joint Space (p < 0.001)** | Healthy | Diabetic | All IWAs (Res) | Non-diabetic IWAs (Res) | Diabetic IWAs (Res) |
| Healthy |  |  |  |  |  |
| Diabetic | 0.2895 |  |  |  |  |
| All IWAs (Res) | **< 0.001** | **0.002** |  |  |  |
| Non-diabetic IWAs (Res) | **0.004** | **0.014** | 0.348 |  |  |
| Diabetic IWAs (Res) | **0.004** | **0.014** | 0.348 | 0.350 |  |
| **Femoral Neck Width (p = 0.023)** | Healthy | Diabetic | All IWAs (Int) | Non-diabetic IWAs (Int) | Diabetic IWAs (Int) |
| Healthy |  |  |  |  |  |
| Diabetic | 0.370 |  |  |  |  |
| All IWAs (Int) | **0.032** | **0.008** |  |  |  |
| Non-diabetic IWAs (Int) | 0.083 | 0.065 | 0.476 |  |  |
| Diabetic IWAs (Int) | 0.056 | **0.010** | 0.476 | 0.459 |  |
| **Muscle Area (p = 0.013)** | Healthy | Diabetic | All IWAs (Int) | Non-diabetic IWAs (Int) | Diabetic IWAs (Int) |
| Healthy |  |  |  |  |  |
| Diabetic | **0.032** |  |  |  |  |
| All IWAs (Int) | **0.040** | 0.453 |  |  |  |
| Non-diabetic IWAs (Int) | 0.120 | 0.473 | 0.439 |  |  |
| Diabetic IWAs (Int) | 0.065 | 0.477 | 0.447 | 0.453 |  |
| **Muscle Area (p= 0.011)** | Healthy | Diabetic | All IWAs (Res) | Non-diabetic IWAs (Res) | Diabetic IWAs (Res) |
| Healthy |  |  |  |  |  |
| Diabetic | **0.032** |  |  |  |  |
| All IWAs (Res) | **0.003** | 0.106 |  |  |  |
| Non-diabetic IWAs (Res) | **0.027** | 0.270 | 0.321 |  |  |
| Diabetic IWAs (Res) | **0.010** | 0.103 | 0.344 | 0.278 |  |
| **Proximal Femoral Shaft: Mean Thick 2d (p = 0.027)** | Healthy | Diabetic | All IWAs (Res) | Non-diabetic IWAs (Res) | Diabetic IWAs (Res) |
| Healthy |  |  |  |  |  |
| Diabetic | 0.685 |  |  |  |  |
| All IWAs (Res) | **0.022** | **0.010** |  |  |  |
| Non-diabetic IWAs (Res) | **0.049** | **0.028** | 0.475 |  |  |
| Diabetic IWAs (Res) | **0.065** | **0.038** | 0.525 | 0.579 |  |
| **Middle Femoral Shaft: Max Thick 2d (p = 0.010)** | Healthy | Diabetic | All IWAs (Int) | Non-diabetic IWAs (Int) | Diabetic IWAs (Int) |
| Healthy |  |  |  |  |  |
| Diabetic | 0.485 |  |  |  |  |
| All IWAs (Int) | 0.010 | 0.014 |  |  |  |
| Non-diabetic IWAs (Int) | 0.163 | 0.195 | 0.146 |  |  |
| Diabetic IWAs (Int) | **0.001** | **0.002** | 0.147 | **0.037** |  |
| **Middle Femoral Shaft: Mean Thick 2d (p = 0.022)** | Healthy | Diabetic | All IWAs (Int) | Non-diabetic IWAs (Int) | Diabetic IWAs (Int) |
| Healthy |  |  |  |  |  |
| Diabetic | 0.370 |  |  |  |  |
| All IWAs (Int) | 0.038 | 0.022 |  |  |  |
| Non-diabetic IWAs (Int) | 0.384 | 0.297 | 0.212 |  |  |
| Diabetic IWAs (Int) | **0.004** | **0.002** | 0.212 | 0.111 |  |
| **Distal Femoral Shaft: SD Thick 2d (p = 0.025)** | Healthy | Diabetic | All IWAs (Res) | Non-diabetic IWAs (Res) | Diabetic IWAs (Res) |
| Healthy |  |  |  |  |  |
| Diabetic | 0.264 |  |  |  |  |
| All IWAs (Res) | 0.044 | 0.067 |  |  |  |
| Non-diabetic IWAs (Res) | **0.007** | **0.007** | 0.126 |  |  |
| Diabetic IWAs (Res) | 0.469 | 0.469 | 0.084 | **0.029** |  |

Supplemental Table 7: Mann-Whitney Comparisons for significant Kruskal-Wallis p-values. The significance level was set α ≤ 0.05. Abbreviations: IWAs= individuals with lower-limb amputations, Int= intact limb, Res= residual limb.
